# Development and Pilot Testing of an Addiction Clinic–Based Pre-Exposure Prophylaxis Uptake and Adherence Intervention for Women with Substance Use Disorders: Protocol for a Pilot Randomized Trial

**DOI:** 10.2196/64961

**Published:** 2025-05-23

**Authors:** Angela M Heads, Diane Santa Maria, Mandy J Hill, Robert Suchting, Kimberly N Evans, Zaneta Gaul, Luba Yammine, Constanza de Dios, Joy M Schmitz

**Affiliations:** 1 McGovern Medical School University of Texas Health Science Center at Houston Houston, TX United States; 2 Cizik School of Nursing University of Texas Health Science Center at Houston Houston, TX United States; 3 National Center for HIV, Viral Hepatitis, STD, and TB Prevention Centers for Disease Control and Prevention Atlanta, GA United States; 4 DLH Corporation Atlanta, GA United States

**Keywords:** HIV prevention, Pre-Exposure Prophylaxis, substance use disorder, clinic-based intervention, harm reduction

## Abstract

**Background:**

Black and Hispanic women in the United States continue to bear disproportionate incidence of HIV related to sexual transmission and injection drug use. Specifically, women with substance use disorders (SUDs) are more likely to engage in vaginal or anal condomless sex associated with HIV transmission. Pre-exposure prophylaxis (PrEP) is a highly effective HIV prevention tool but is not widely used by racial or ethnic minority women. Effective interventions for engaging women with SUDs in HIV prevention interventions that are culturally appropriate and, therefore, more appealing to racial or ethnic minority women with SUDs are critically needed.

**Objective:**

This 3-phased study, including a pilot randomized controlled trial (RCT), will assess the initial efficacy, feasibility, and acceptability of an addiction clinic–based behavioral and PrEP services intervention to increase the uptake and adherence to PrEP among racial or ethnic minority women.

**Methods:**

A 3-phased mixed methods research design will involve formative qualitative methods using thematic analysis to design the intervention (phase 1), theatre testing to adapt and refine the intervention (phase 2), and RCT methods to pilot test the intervention for efficacy, feasibility, and acceptability (phase 3). The pilot RCT will enroll and randomize 60 women to either the standard SUD treatment program or SUD treatment integrated with PrEP services. The addiction clinic–based behavioral intervention will include 4 motivational counseling sessions guided by the Information-Motivation-Behavioral Skills Model to increase the uptake of PrEP. A mobile health app will be used to engage participants with the intention of motivating PrEP initiation and supporting adherence to PrEP. Following phase 3, generalized linear modeling will be used to model effects of the proportion of participants who fill their prescription and take at least 1 dose as a function of the intervention group.

**Results:**

Findings from individual qualitative interviews informed the development of the addiction clinic–based behavioral intervention. Study recruitment for the randomized pilot (phase 3) launched in May 2024. Additional statistical analyses will be performed upon completion of the study.

**Conclusions:**

This addiction clinic–based behavioral intervention aims to increase PrEP uptake and adherence among racial or ethnic minority women who engage in sexual and substance use behaviors associated with increased susceptibility to HIV transmission. The addiction clinic–based behavioral intervention has the potential to reduce HIV-related disparities among Black and Hispanic women with SUDs. Findings from this study will provide a foundation for future HIV prevention interventions for racial or ethnic minority women with SUDs.

**Trial Registration:**

ClinicalTrials.gov NCT06158607; https://clinicaltrials.gov/study/NCT06158607?term=NCT06158607&rank=1

**International Registered Report Identifier (IRRID):**

DERR1-10.2196/64961

## Introduction

### Background

In the United States, women with a lower socioeconomic status and women from racial or ethnic minority backgrounds continue to bear a disproportionate HIV burden [1]. According to the Centers for Disease Control and Prevention (CDC), women made up 18% of the new HIV diagnoses in 2022, with 83% of those cases representing transmission through heterosexual contact and 16% representing transmission through injection drug use. The HIV diagnosis rates for Black and Hispanic women are 10 and 3 times, respectively, the rates for White women [1]. Women with substance use disorders (SUDs) are particularly vulnerable to HIV transmission due to the greater likelihood of engaging in vaginal or anal sex without using a condom, concurrent sexual relationships, sex in exchange for drugs, money, or other items, and sexual coercion from experiencing sexual abuse or assault by intimate partners [2-6]. Inequities experienced by women, including relationship power and control differentials and physical or sexual violence, further influence women’s engagement in both the risk behaviors that increase their likelihood of contracting HIV as well as the potential preventive measures. To adequately address the syndemic effect of substance use, HIV, and trauma [7], interventions must be specifically developed for the unique needs of this population.

Pre-exposure prophylaxis (PrEP) is a medication with demonstrated efficacy in reducing HIV transmission, including among heterosexual women [8]. PrEP removes the barrier of partner consent, thus empowering women to engage in their own HIV prevention strategies. This is an important factor among women who have difficulty negotiating safer sex practices with their partners due to relationship power imbalances [9-11]. Despite the effectiveness of PrEP, uptake has been slow among women, and the reasons for this, particularly among women with SUDs, are not completely understood [12].

According to the CDC, in 2019, approximately 1.2 million adults had PrEP indications. Yet, there are disparities among those with PrEP indications, with males more likely to have been prescribed PrEP than females [13]. HIV prevention research has overwhelmingly focused on men who have sex with men, with few studies designed specifically to study PrEP uptake in women [14,15]. Evidence suggests that low perceived risk and concerns about side effects (most commonly nausea and vomiting) are barriers to PrEP use [16]. Some women perceive themselves to be at low risk for HIV despite engaging in condomless sex and sex while using substances [17-19]. In contrast, studies examining PrEP knowledge and attitudes have found low rates of initial knowledge with high levels of interest among women once they are informed of the benefits of PrEP for HIV prevention [14,15]. This interest, however, does not always result in PrEP uptake.

Social and structural factors such as poverty, race or ethnicity, inequities, discrimination, and lack of empowerment combine as barriers to HIV prevention for women with SUDs [9-11,20-25]. Extending availability and access to effective HIV prevention strategies, such as PrEP, may help overcome these intersecting barriers and is consistent with the nation’s “Ending the HIV Epidemic” plan by “preventing new HIV transmissions by using effective interventions which include PrEP” [26]. In addition to the individual barriers to PrEP use mentioned above, there are also social and structural barriers to PrEP uptake. These include, but are not limited to, perceived stigma, lack of access to knowledgeable providers, and mistrust of health care providers and health care systems [12,14,15,27]. Lack of access to PrEP services and provider reluctance to prescribe PrEP are also significant barriers, given that women have reported a preference for conversations about HIV prevention to be initiated by medical providers [17]. Expanding PrEP uptake to women is a vital component of the nation’s plan [28]. However, research on PrEP initiation among women is limited [29].

A few studies have found daily oral PrEP to be effective for reducing HIV acquisition in heterosexual women [8,30]. However, 2 major trials specifically examining PrEP in women were unable to demonstrate the efficacy of oral PrEP because of low adherence [31,32]. The few published research studies on PrEP uptake among women have taken place in a variety of US and international settings where available resources and barriers to uptake and adherence may vary considerably, thus making it difficult to ascertain the potential for PrEP to reduce HIV acquisition among heterosexual women. The notable absence of published findings on effective interventions to increase PrEP uptake and adherence among women represents a significant gap in the research literature [33]. A recent review of best practices for increasing PrEP uptake and persistence in the United States identified 9 interventions meeting the criteria for best practice, with only 1 of the 9 including women as participants [34]. The 1 study included in the review, which included women, reported only 6 of the 198 participants identified as women [35]. One promising intervention using motivational interviewing with women was not included as meeting best practice guidelines due to low participant enrollment [36]. Additional research on interventions designed to increase PrEP uptake and adherence among women is needed [15]. A meta-analysis of oral PrEP use among women found only a 61% reduction in HIV acquisition risk among women who reported 75% adherence to a daily PrEP regimen [37]. Researchers have called for greater attention to both PrEP uptake and adherence among women and future studies that focus on developing and evaluating methods to enhance adherence and greater reliance on laboratory-based versus self-reported adherence measures [38].

Integrating HIV prevention into environments where individuals are already engaged in other services helps to increase access to care. In a previous study, researchers found that offering HIV testing and prevention counseling within SUD treatment programs resulted in increased rates of receiving these preventive services [39]. This is an example of work at an individual level to increase HIV testing behavior and at a structural level to remove barriers to care by increasing the SUD treatment program’s capacity to provide HIV prevention services [40]. It has been recommended that SUD treatment programs routinely offer HIV testing to all participants and linkage to primary medical care for individuals who test positive for HIV as a means of increasing access to HIV care [41]. However, despite the well-known associations among substance use, HIV, and sexual risk, it is estimated that only about half of SUD treatment programs offer HIV prevention services [42,43].

The addiction treatment clinic setting is ideally situated to provide additional services for HIV prevention. In recognition of the important role that addiction providers can play in the adoption and delivery of PrEP services and to identify potential barriers to implementing PrEP, researchers have explored providers’ perspectives on including PrEP as part of addiction treatment [44]. To our knowledge, PrEP and HIV prevention services have not been optimally integrated into the addiction treatment clinic environment; thus, the study team proposed to develop an addiction clinic–based behavioral intervention designed to increase PrEP uptake for racial or ethnic minority women.

### Theoretical Framework

**Figure 1:**
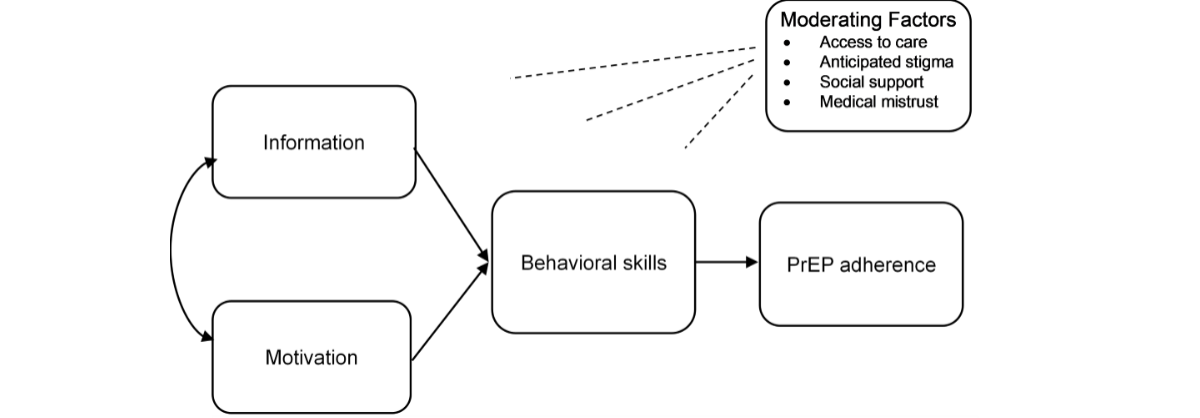
The Information-Motivation-Behavioral Skills model of pre-exposure prophylaxis uptake (adapted from Fisher et al [47]). PrEP: pre-exposure prophylaxis.

This project is guided by the Information-Motivation-Behavioral Skills (IMB) [45] model, shown in Figure 1. The IMB model of health behavior change posits that information, motivation, and behavioral skills are key determinants of engaging in health behaviors. This model has been applied to various health promotion behaviors, including HIV treatment and prevention [40-43]. Specific to HIV prevention, the 3 fundamental determinants of HIV risk reduction include information regarding how HIV is transmitted and methods for preventing HIV infection; motivation to change risky behaviors; and training in behavioral skills necessary for engaging in specific preventive acts [46]. In HIV research, the IMB model has been used to inform interventions for medication adherence in persons living with HIV [47] and has some preliminary evidence for utility with PrEP uptake and adherence interventions [48-50]. The IMB model has also been used to guide the development of interventions to increase PrEP uptake and adherence using mobile health (ie, text messaging, appointment reminders, and medication reminders using mobile devices) [51,52]. Previous research supports the use of mobile technologies as a cost-effective and convenient method for increasing health-promoting behaviors [53].

Research supports the IMB model in the development of interventions aimed at increasing willingness to use PrEP in people who use drugs with high HIV risk [54]. Guided by the IMB model, our addiction clinic–based PrEP uptake and adherence intervention provides knowledge of PrEP effectiveness and side effects and offers motivational incentives for increasing PrEP uptake using an innovative remote-delivery monetary incentive application (Scene mobile app; formerly Emocha [55,56]) that provides the behavioral skills necessary to effectively use PrEP (eg, adherence and side effect management) including video directly observed therapy to monitor medication taking. Video directly observed therapy allows participants to upload videos of themselves taking their medication through a video-enabled device such as a smartphone so that their treatment teams can verify that medication has been taken. In addition, the Scene Health mobile app includes videos showing behavioral skills, a library of motivational messages, and frequently asked questions to address barriers to PrEP use.

Within the context of the IMB model, the research team anticipates that the addiction clinic–based behavioral intervention will improve PrEP uptake and adherence among racial or ethnic minority women engaged in SUD treatment. By co-locating services within the addiction clinic, the intervention will address moderating factors (access to care and adherence support) that have been barriers to successful PrEP uptake and adherence.

### Objectives and Study Design

**Figure 2:**
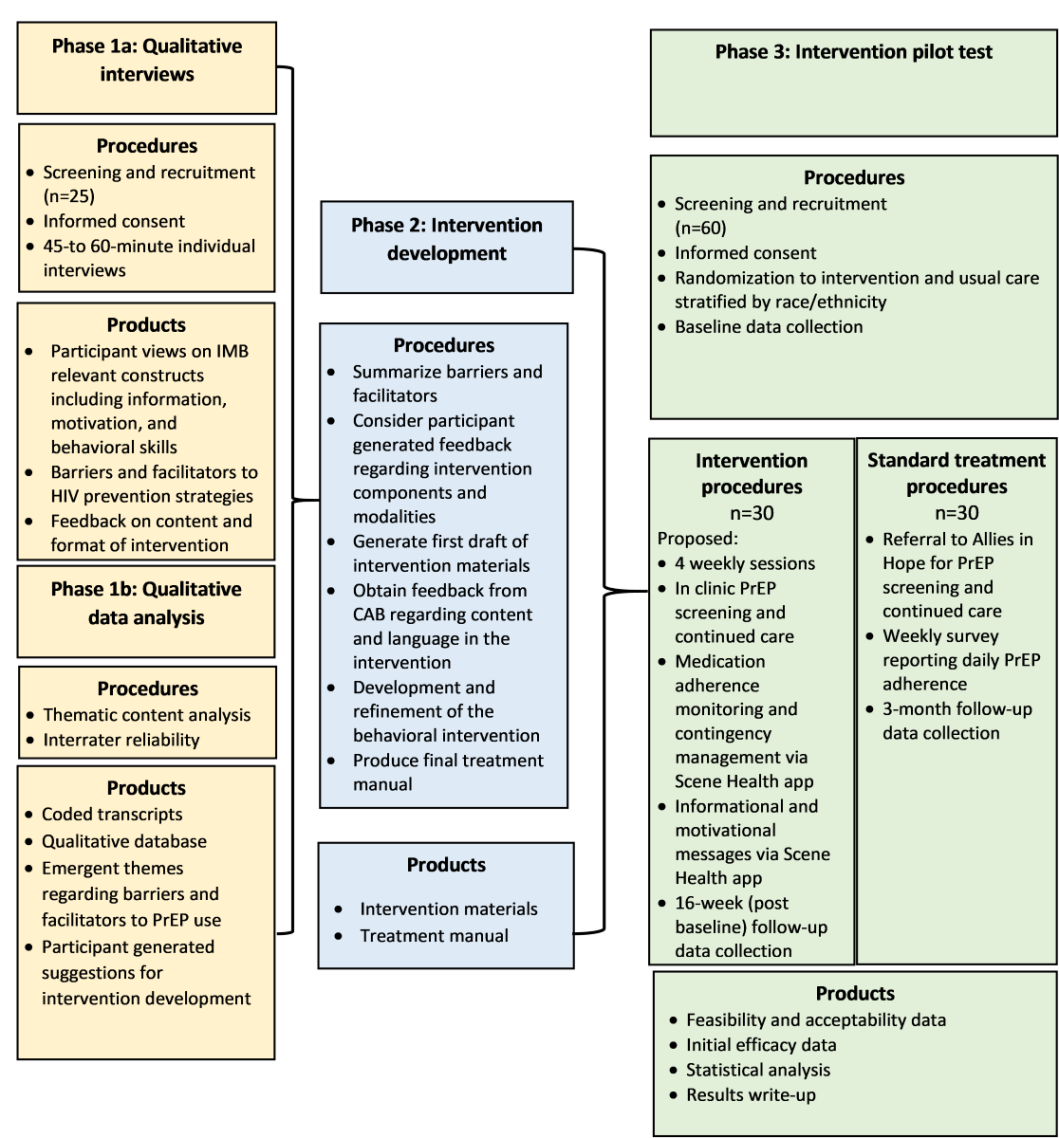
Overall study design. Development and pilot testing of an addiction clinic–based prep adherence intervention for women with substances use disorders. CAB: community advisory board; IMB: Information-Motivation-Behavioral Skills: PrEP: pre-exposure prophylaxis.

This study aims to develop an addiction clinic–based behavioral intervention for increasing PrEP uptake and adherence among racial or ethnic minority women who engage in sexual and substance use behaviors associated with HIV transmission. To accomplish this, investigative team proposes a 3-phase study, as illustrated in Figure 2, to address 3 specific aims: (1) to elicit information on knowledge and attitudes about PrEP use and obtain feedback about the design of an addiction clinic–based behavioral intervention for racial or ethnic minority women with sexual and substance use behaviors associated with HIV transmission; (2) to develop an addiction clinic–based behavioral intervention to promote PrEP uptake and adherence in women undergoing treatment for SUD who are susceptible to HIV infection; and (3) to assess initial efficacy, feasibility, and acceptability of the addiction clinic–based behavioral intervention.

## Methods

### Study Setting

The primary study setting will be the outpatient treatment research clinic of the Center for Neurobehavioral Research on Addiction (CNRA), a university-supported center of excellence within the Louis A Faillace, MD Department of Psychiatry and Behavioral Sciences at UTHealth Houston. Allies in Hope (formerly AIDS Foundation Houston), established in 1982, is a community-based organization providing HIV prevention, HIV treatment, counseling, and case management to Houston residents. Allies in Hope will be the referral site for the control group (ie, standard-of-care treatment condition) in the phase 3 pilot randomized controlled trial.

### Study Population and Eligibility

Participants are eligible to participate in all phases of the study if they are self-identified as Black/African American and Hispanic/Latina women, aged 18 years or older, diagnosed with an SUD per *DSM-5* (*Diagnostic and Statistical Manual of Mental Disorders* [Fifth Edition]) criteria [57], HIV-negative, sexually active (ie, vaginal or anal sex) with a male within the past 6 months, not using PrEP for HIV prevention at the time of screening, fluent in English, and own or have regular access to a smartphone.

Participation in the current study is limited to English-speaking individuals because intervention materials have not yet been translated into Spanish. Future research will use instruments translated into Spanish and include interview and data collection materials in Spanish to address the unique needs of individuals whose primary or preferred language is Spanish.

### Phase 1: Formative Research

#### Phase 1a: In-Depth Qualitative Interviews

This phase of the study was conducted between June 2022 and February 2023. We recruited 25 women to complete in-depth qualitative interviews to provide their personal and detailed perspectives about PrEP and our proposed addiction clinic–based behavioral intervention. Recruited women were currently enrolled in treatment for SUD. The interviews lasted between 45 to 60 minutes, and participants were compensated US $25 for their participation.

The interview guide was developed based on the IMB model, focusing on the following areas:

1. Information: participants’ knowledge and awareness of PrEP, including their understanding of its effectiveness, who it is recommended for, the importance of taking it consistently, potential side effects, and possible drug interactions.
2. Motivation: participants’ attitudes toward HIV prevention and PrEP, perceived barriers to using PrEP such as beliefs about its outcomes, experiences or concerns related to stigma, and the level of social support from partners, family, and friends.
3. Behavioral skills: participants’ confidence in their ability to prevent HIV, strategies for retaining HIV prevention information, maintaining medication adherence (eg, setting reminders), attending follow-up medical appointments, and managing side effects.

The interviews also gathered insight about barriers and facilitators of the proposed integration of HIV prevention services within standard SUD treatment, specifically (1) preferences for in-person versus remote delivery of motivational enhancement interventions to improve PrEP adherence, (2) frequency and duration of sessions; (3) frequency of motivational messages, and (4) content of motivational messages. Interviewers also introduced the Scene Health app and obtained feedback on the appointment reminder, video uploads for medication adherence, and notification of the app’s awards (ie, monetary incentives) features.

A community advisory board (CAB) consisting of 6 members from local community-based organizations who are representative of the populations of interest for the current study was created. Members of the CAB provided feedback on surveys, interview guides, advertising, and other study materials. CAB member feedback on the motivational messaging included in the intervention was also elicited.

#### Phase 1b: Data Analysis

Qualitative data were analyzed using MAXQDA. Our analysis used a thematic analysis approach, which involves identifying themes through an iterative process comprised of multiple stages of data collection, theme refinement, and data categorization. The investigators used the IMB framework to organize emergent themes based on participants’ interviews. The team used the results from the qualitative data analysis to inform the development of the addiction clinic–based behavioral intervention. Future manuscripts will present findings from phase 1 of the study.

### Phase 2: Development and Theater Testing of the Cultivating HeAlth Resilience Growth & Empowerment (Take CHARGE) Intervention: Addiction Clinic–Based Behavioral Intervention

Based on information obtained in phase 1 and the IMB model, the study team developed an addiction clinic–based behavioral intervention, Theater Testing of the Cultivating HeAlth Resilience Growth & Empowerment (Take CHARGE), that provides PrEP-related information, motivational messaging, and behavioral skills necessary to use PrEP effectively. The intervention will include a PrEP service component to be delivered in the addiction treatment clinic by a nurse practitioner, leveraging the existing SUD treatment and HIV prevention infrastructure and removing accessibility barriers. This intervention will be provided alongside standard SUD treatment which consists of participation in individually delivered, evidenced-based intervention (eg, cognitive behavioral therapy) focused on quitting or reducing substance use delivered by a master’s-level counselor, psychologist, or psychology intern under the supervision of a licensed psychologist.

The intervention will consist of 4 weekly sessions that can be delivered through a HIPAA (Health Insurance Portability and Accountability Act)-compliant videoconferencing platform. Sessions will be approximately 30 minutes in length and are intended to increase knowledge of PrEP, increase positive attitudes toward PrEP, and decrease behaviors that place individuals at risk for HIV infection with a goal of increasing PrEP uptake and PrEP adherence.

### The IMB Framework

The study team used the IMB domains to develop the addiction clinic–based behavioral intervention. Specifics on how each domain informed the development of the intervention are outlined below.

#### The Information Domain

The information component involved providing basic information about PrEP, including how it works, who is eligible, safety and efficacy, and the importance of adherence. The information component also provided information about the benefits of individually controlled HIV prevention methods (eg, not relying on men to wear condoms, racial or ethnic minority women maximizing their power and decision-making abilities for their sexual health) [50]. Based on findings in Phase 1, the study team tailored the intervention to address gaps in knowledge and misconceptions about HIV and PrEP.

#### The Motivation Domain

The motivational enhancement component included motivational counseling and monetary incentives for achieving desired behaviors. To address low perceptions of HIV risk as a barrier, the intervention will individually address perceived versus actual personal risk for HIV infection. The motivational therapy will address attitudes about the potential advantages of using PrEP and potential outcomes of not using PrEP. To address the social aspects of motivation, sessions will explore stigma and social support concerns. Monetary incentive-based motivation will be embedded into video-based directly observed therapy using the Scene Health mobile app and is a CDC-endorsed model of care to ensure medication adherence while capturing adverse events [51,52]. The platform will be customized to provide appointment reminders and immediate delivery of monetary incentives (refer to the Monetary Incentives subsection under Intervention Arm and Control Arm subsection) upon confirmation of engagement in the target behavior (ie, attendance and medication taking).

#### The Behavioral Skills Domain

The behavioral skills development component will provide specific skills to increase self-efficacy. This included helping the participant identify ways to remind themselves to take their medication, communicating a need for social support, managing side effects, and identifying supportive others. This component leverages the SUD treatment clinic’s current HIV prevention infrastructure to deliver PrEP services including initial screening, prescription, side effect monitoring, and follow-up to women engaged in SUD treatment. The study team proposes to fully integrate PrEP services into the addiction clinic setting. All services, including laboratory tests, monitoring, and follow-up, will be delivered within the clinic setting for those in the intervention arm. Individuals in the control arm of the study will be referred to Allies in Hope for standard-of-care PrEP services. They will be provided with an appointment and transportation assistance. Participants in both conditions will continue receiving their addiction treatment services.

#### Motivational Messages

The study team also created a library of approximately 200 motivational messages to be delivered between sessions. These will include text-based messages and brief videos created by research staff, adapted from the UCARE4LIFE study [58], and supplemented with the CDC’s #ShesWell: PrEP for Women campaign to promote the uptake of PrEP among women of color. A sample of these messages were provided to phase 1 and phase 2 participants who provided their feedback on the language and acceptability of the messages. The messages are intended to motivate participants to attend their appointments with the nurse practitioner, take PrEP daily as prescribed for those on daily oral PrEP, continue their SUD treatment, speak with the nurse practitioner regarding side effects or questions, and decrease sexual behaviors that may increase their susceptibility to HIV infection.

#### Theater Testing

The investigators invited 5 participants who had completed the qualitative interviews in phase 1 to a theater testing session to provide feedback on the developed intervention, including the motivational messages that had been created. The investigators conducted theater testing from February 19 to February 28, 2024. Participants were compensated US $25 for their time. During the theater test, participants completed a 45-minute sample session, which included elements from the 4 planned sessions of the full intervention. Following the theater test, each participant completed a feedback form with open and closed-ended questions and participated in a 15-minute interview to obtain their reactions to the proposed intervention [59]. The research team used this feedback to tailor the specific content of the sessions further to be more appealing to women engaged in treatment for SUD who have increased susceptibility to acquiring HIV.

### Phase 3: Intervention Pilot Test—Study Design

The investigative team will rigorously pilot test the degree to which the addiction clinic–based behavioral intervention promotes increased PrEP adherence compared with standard linkage to PrEP. The investigative team will randomly assign 60 women to 1 of the 2 conditions. Participants in both the intervention and control groups will continue to receive evidence-based behavioral therapy for SUDs (ie, cognitive behavioral therapy with licensed counselors).

### Participants and Recruitment

The investigators will recruit 60 BlackBlack or Hispanic women to participate in the study. The investigators will invite individuals who are engaged in or initially enrolling in treatment for SUDs at the CNRA. The research assistant will describe the study by informing participants that this is an HIV prevention study focusing on women engaged in treatment for SUD and will not directly impact their regular SUD treatment. Potential participants will be screened over the phone by the research assistant and informed of the purpose of the study. Those meeting inclusion criteria and agreeing to participate will sign an informed consent form and receive a copy. After completing the informed consent process, participants will be randomized to the intervention or control group.

The study team will recruit eligible participants by posting flyers, through word of mouth (through addiction treatment staff referral), and advertising in local print sources (newspapers and magazines). Recruitment and enrollment began in May 2024 and will continue through March 2025.

### Intervention Arm

#### Weekly Counseling Sessions

Participants randomized to the addiction clinic–based behavioral intervention will receive 4 motivational counseling sessions (~30 minutes each) related to HIV prevention, PrEP, and partner communication information delivered by a trained study counselor with expertise in HIV prevention among substance using populations. The study counselor will assist the participant with downloading the Scene app and completing a profile. The incentive scheme and expectations for treatment will be explained at the time of the baseline assessment.

#### In-Clinic PrEP Screening

After completion of the four motivational sessions, the participant will be scheduled for their initial visit with the nurse practitioner in the CNRA, who will conduct a clinical assessment which will include fourth-generation HIV screening (ie, tests that detect both HIV antibodies and p24 antigens), screening for sexually transmitted infections, a basic metabolic panel to include serum creatinine, and hepatitis B and C screening. The nurse practitioner will review laboratory results. If there is no contraindication for PrEP, the participant will be provided a prescription for PrEP. The decision to prescribe daily oral PrEP or long-acting injectable (LAI) PrEP will be based on a collaborative decision-making process between the patient and nurse practitioner, considering clinical appropriateness, patient choice, and medication availability. Participants on daily oral PrEP will be instructed to take the medication daily and to follow up for laboratory tests in 12 weeks while participating in the intervention. Participants enrolled in LAI-PrEP will be provided with a prescription for a 4-week supply of oral cabotegravir per local clinic protocol of prescribing lead-in medication to monitor side effects before initiating LAI-PrEP. Participants are then instructed to follow up with the nurse practitioner in 4 weeks for their first injection while participating in the intervention. All participants who receive a PrEP prescription from the on-site nurse practitioner will meet with the risk reduction specialist, who will assist those without prescription insurance with enrollment for an appropriate patient assistance program to access medication free of charge through the Advancing Access Program or the Health and Human Services ready, set, PrEP program.

#### Medication Adherence Monitoring

Participants will be instructed to provide proof that they are in receipt of the prescribed PrEP medication (video message within the Scene app) and to begin daily video uploads of adherence to medication regimen. Participants who do not fill their PrEP prescription within 2 weeks will be scheduled for a follow-up visit with the nurse practitioner, who will address any concerns and questions related to starting the medication. Participants who are prescribed daily oral PrEP will be asked to upload daily adherence videos (ie, video of the participant taking the medication) for 12 weeks after initiating PrEP.

#### Motivational Messaging

Participants will receive motivational messages through text for the entire 12 weeks to promote medication uptake (for participants who, after the motivational counseling sessions, have not chosen to start PrEP and agree to continue receiving the informational messages) or reminders and motivational messages to support adherence to PrEP (for participants who initiate PrEP). All participants will be given the option of choosing how often they receive the messages and can opt out of messaging at any time through the app.

#### Monetary Incentives

Participants will receive monetary incentives for completing project-related tasks and engaging in desirable health-promoting behaviors. Each participant will receive US $25 for the baseline data collection, US $10 per session for attending 4 weekly motivational counseling sessions, US $25 for the initial nurse visit with laboratory tests, US $10 for providing proof of the initial prescription fill, US $1/day for uploading daily adherence videos during weeks 5-16 (those on LAI-PrEP will receive US $1/day for weeks 4-8 and US $60 for proof of injection for weeks 9-16), US $25 each for data collection at 4-weeks and 1-weeks post baseline, US $25 for the 90-day follow-up visit with the nurse practitioner, and US $50 for completing the follow-up data collection at week 16 (US $309 possible for the entire intervention).

#### Control Arm

Participants randomized to the control arm will receive standard of care PrEP information along with a referral and a scheduled appointment with Allies in Hope. Transportation assistance for appointment attendance will be provided. In this agency, if there is no contraindication for PrEP, the participant will be provided a prescription for daily oral PrEP or long-acting injectable PrEP and follow up with the community-based partner per normal procedures at that facility. Allies in Hope will provide the research team with information regarding appointment attendance and whether a prescription was provided to the participant, with consent from the participant. Participants in standard treatment will continue to receive their regular SUD treatment services.

#### Monetary Incentives

Participants in the control arm will receive the same incentives, except for the weekly session amounts, for a possible total of US $269 for the entire study. Standard treatment participants will not have access to the Scene Health app and will be asked to respond to a daily yes or no question regarding medication adherence through REDCap (Research Electronic Data Capture; Vanderbilt University) with the same US $1/response incentive.

### Data Collection

Participants in both arms will complete baseline surveys and follow-up surveys administered at 4 weeks, 10 weeks, and 16 weeks post baseline. Data sources to examine efficacy will include a computer-assisted self-interview (self-report measures), pharmacy records, Scene Health app data, and medical records.

Master’s-prepared clinicians (eg, mental health counselors and psychologists) will conduct a comprehensive diagnostic interview using the Mini International Neuropsychiatric Interview (MINI) [60] and the National Institute on Drug Abuse Modified Assist [61]. This will be used to determine a diagnosis and severity of SUD and any co-occurring mental health disorders. Other data collection will include baseline assessment of outcome variables and potential moderators. These self-report questionnaires will be delivered through a REDCap survey. The link will be emailed or texted to the participant based on their preference. Refer to Table 1 for a description of measures and outcomes of interest.

**Table 1:** Overview of phase-wide data collection methods and measures-Harris County, Texas (2021-2025).

Construct			Measure		Study week		Source
**Eligibility**							
	SUD^a^	Substance use and co-occurring mental health diagnoses (Mini International Neuropsychiatric Interview)		0		SUD intake records	
**PrEP knowledge and attitudes (based on barriers identified in aim 1)**							
	PrEP^b^ knowledge	PrEP knowledge questionnaire [62]		0, 4, 10, 16		REDCap^c^ survey	
	PrEP attitudes	PrEP Attitudes Measure [63]		0, 4, 10, 16		REDCap survey	
	PrEP stigma	PrEP Anticipated Stigma Scale (PASS) [64]		0, 4, 10, 16		REDCap survey	
	PrEP adherence self-efficacy	Adherence Starts with Knowledge (ASK-20) [65]		0, 4, 10, 16		REDCap survey	
	HIV risk behaviors	Risk Assessment Battery [66]		0, 4, 10, 16		REDCap survey	
	HIV risk perceptions	Perceived Risk of HIV Scale [67]		0, 4, 10, 16		REDCap survey	
**Initial efficacy (aim 3)**							
	PrEP eligibility	HIV screening, viral hepatitis screening, pregnancy screening, screening for sexually transmitted diseases, and behavioral risk factors		4, 16, 4		Laboratory results Nurse interview	
	PrEP uptake (primary outcome)	PrEP prescription filled and self-reported initial dose of PrEP taken		10, 16		Self-report	
	PrEP adherence (behavioral)	Video-confirmed dose (intervention, daily oral PrEP) Video-confirmed dose (intervention, long-acting injectable [LAI] PrEP, and initial oral cabotegravir) Follow-up injection (intervention LAI) Self-reported dose daily oral PrEP (intervention and standard treatment)		5-16 n/a and 5-16		Scene health reports, study records, study records, and weekly online survey	
	PrEP adherence (laboratory-confirmed)	Tenofovir concentrations (>1000 ng/mL)		16		Laboratory results	
**Feasibility measures (aim 3)**							
	Enrollment rate	Proportion screened who are eligible Proportion eligible who consent		—^d^		Study records	
	Session attendance	Proportion of motivational enhancement sessions attended		—		Study records	
	Use of Scene Health app	Proportion of medication adherence videos uploaded		—		Scene Health reports	
	Study retention	Proportion of participants who remain in the study at 12-week follow-up		—		Study records	
**Acceptability measures (aim 3)**							
	Client satisfaction	Client satisfaction questionnaire		4, 16		REDCap survey	
**Other covariates**							
	Substance use severity	NIDA-Modified Alcohol, Smoking, and Substance Involvement Screening Test [68]		0, 4, 10, 16		REDCap survey	
	Alcohol	Alcohol Use Disorders Identification Test [69]		0, 4, 10, 16		REDCap survey	
	Health literacy	Calgary Charter on Health Literacy Scale [70]		0		REDCap survey	
	Medical mistrust	Group Based Medical Mistrust Scale [71]		0, 4, 10, 16		REDCap survey	
	Trauma	Life Events Checklist/PTSD^e^ Checklist for *DSM-5*^f^ [72,73]		0, 4, 10, 16		REDCap survey	
	Depression	Patient Health Questionnaire-9 [74]		0, 4, 10, 16		REDCap survey	
	SUD treatment attendance	Number of substance use disorder therapy sessions attended		16		Study records	

^a^SUD: Substance use disorder.

^b^PrEP: pre-exposure prophylaxis.

^c^REDCap: Research Electronic Data Capture.

^d^Not applicable.

^e^PTSD: posttraumatic stress disorder.

^f^*DSM-5: Diagnostic and Statistical Manual of Mental Disorders* (Fifth Edition).

### Primary Outcome

The primary outcome of interest is PrEP uptake defined as filling the PrEP prescription and taking at least 1 dose of the medication by 2 weeks following their visit with the PrEP provider. The primary outcome will be measured at 12 weeks post completion of the 4-week motivational sessions.

### Secondary Outcome

The secondary outcome of interest is daily PrEP adherence as measured by the proportion of videos uploaded showing medication being taken (intervention group only), the proportion of self-reported daily adherence (intervention and control group), levels of tenofovir (TFV) urine concentrations (>1000 ng/mL) [75] detected in urine measured at 12-weeks post-PrEP initiation (both groups). According to previous research, urine concentrations of TFV at >1000 ng/mL is indicative of use of oral PrEP in the previous 3 days (high adherence), detectable levels of TFV but <1000 ng/mL are indicative of low adherence and undetectable levels of TFV suggesting nonadherence [75]. Studies have also shown that directly observed therapy is useful for interpreting PrEP adherence. This provides an additional layer of certainty regarding this method of measuring this important secondary outcome [76]. Medication adherence for long-acting injectable PrEP will be determined by examining medical records for the timing of the dose.

### Other Outcomes

Other outcomes of interest include intervention feasibility (enrollment rate, session attendance, use of Scene Health, and study retention), and intervention acceptability (client satisfaction scores).

### Safety Considerations

A data safety monitoring board affiliated with the CNRA will provide oversight for this study including the possibility of loss of confidentiality. The Scene Health mobile app is HIPAA-compliant and allows for secure messaging and encrypted videos, Access to the app requires a participant to log in with credentials. Videos submitted to the app are not available in the device gallery and videos are deleted from the device once received by Scene Health’s secure server. Only approved project staff will have access to the participant data via the “Backstage” by Scene Health web portal.

### Statistical Analysis

Generalized linear modeling will model effects of the proportion of participants who fill their prescription and take at least one dose as a function of intervention group. In unique models, generalized linear modeling will evaluate 2 secondary outcomes as a function of intervention group: (1) dichotomous verification of adherence based on urine TFV levels >1000 ng/mL (no vs yes), and (2) TFV levels >1000 ng/mL indicating recent adherence, detectable levels of TFV but <1000 ng/mL indicating low adherence and undetectable levels of TFV suggesting nonadherence [75].

Where necessary, generalized linear mixed modeling will account for correlated observations by including random effects (eg, random intercepts or slopes for participant ID in longitudinal analyses of adherence over time). Survival analysis (ie, Kaplan-Meier estimation; Cox proportional hazards regression) will evaluate PrEP retention. Additional analyses will evaluate descriptive statistics about the enrollment rate, study retention, session attendance, and participant satisfaction. Analyses will be performed in R (R Foundation for Statistical Computing) [77]. Statistical significance will be measured at an α of <.05 for all a priori hypothesis tests. Hypothesis 3a: (primary outcome) compared with standard treatment, the intervention will result in a greater proportion of participants who fill their first PrEP prescription. Hypothesis 3b: compared to standard treatment, the intervention will result in greater adherence to PrEP in those who filled their prescriptions (self-report and laboratory verified urine tenofovir concentrations). Hypothesis 3c: the intervention will be feasible and acceptable by women as demonstrated by enrollment rate, study retention, session attendance, and participant satisfaction scores.

### Ethical Considerations

This study is approved by the University of Texas Health Science Center at Houston Committee on the Protection of Human Subjects (HSC-MS-21-0451). This study is registered on ClinicalTrials.gov (NCT06158607).

## Results

A total of 25 participants were recruited to complete phase 1, qualitative interviews that informed the final intervention. Findings from phase 1 will be reported in a future manuscript. In October 2023, the investigators received institutional review board approval to conduct theater testing of the intervention (phase 2). Theatre testing has been completed with 5 participants, resulting in final refinements to the intervention. Phase 3 started in May 2024, with preliminary results anticipated by January 2025. The study team plans to publish all findings in future manuscripts and disseminate findings at future conferences and to all community partners. Refer to Figure 2 for the overall study design.

## Discussion

### Principal Findings

The investigators anticipate that the addiction clinic–based behavioral intervention will address the structural and social barriers often cited for their associations with a lack of PrEP uptake and adherence among racial or ethnic minority women. This intervention is innovative in its integration of PrEP services co-located with SUD treatment setting and using mobile health technology to support adherence to PrEP. Although the focus of the intervention is primarily on individual level factors (knowledge, motivation, and skills), there are some aspects of the intervention that help address interpersonal and structural level barriers. The availability of PrEP services within a familiar treatment setting removes a barrier of access for women with substance use disorder. The intervention’s added focus on communication and strategies for seeking social support and knowing when and when not to disclose about PrEP use addresses interpersonal barriers. Through this intervention, the goal is that women will be empowered to engage in their own HIV prevention strategies, promote PrEP as a low-barrier and safe tool for HIV prevention, and remove potential concerns (eg, side effects, confidence in PrEP adherence, stigma from providers, family, and friends) [78,79], to reduce HIV-related disparities in Black and Hispanic women.

### Limitation and Strengths

The current study is primarily limited by scale, given that this is a pilot study of HIV-negative Black and Hispanic women living in Houston, Texas, United States, findings may not be generalizable to areas with divergent demographic characteristics. In addition, participants in the control group will provide self-reported data, which may be subject to recall bias. Due to the limited nature of this pilot study mechanism, it is not possible to follow participants for a longer term as would be necessary to draw conclusions about medication adherence. This will be accomplished through a future larger-scale trial of the intervention. In addition, to test the feasibility and acceptability of the Scene Health app components, we required that participants have regular access to a smartphone. The study team’s experience with this population indicates that the vast majority of participants have access to smartphones. However, this can change on an individual bases as participants have difficulties with paying bills, lose phones, etc. The study team will monitor participants’ access as 1 of the aspects of feasibility for this type of intervention to be further explored in a future implementation study. Finally, participants must speak English to meet eligibility requirements, thus potentially limiting the generalizability of findings for Hispanic women who may only speak Spanish.

Despite these limitations, this study has several strengths. First, there are limited studies specifically focused on developing effective behavioral interventions for women with SUD. Second, tailoring the intervention with the use of videos and content representative of women of color has the potential to engage this population more effectively in care than existing interventions. Third, the intervention uses an app that can help support PrEP adherence by providing information, motivation, and behavioral skills necessary to increase the likelihood of starting and staying on PrEP.

### Conclusions

The addiction clinic–based behavioral intervention aims to increase PrEP uptake and adherence among racial or ethnic minority women who engage in sexual, and substance use behaviors associated with HIV transmission. This addiction clinic–based behavioral intervention has the potential to reduce HIV-related disparities among Black and Hispanic women with SUDs. Findings from this study can also serve as a framework for future culturally appropriate HIV prevention interventions for women.

## Abbreviations

CAB: community advisory board
CDC: Centers for Disease Control and Prevention
CNRA: Center for Neurobehavioral Research on Addiction
*DSM-5*: *Diagnostic and Statistical Manual of Mental Disorders* (Fifth Edition)
HIPAA: Health Insurance Portability and Accountability Act
IMB: Information-Motivation-Behavioral Skills
LAI: long-acting injectable
MINI: Mini International Neuropsychiatric Interview
PrEP: pre-exposure prophylaxis
RCT: randomized controlled trial
REDCap: Research Electronic Data Capture
SUD: substance use disorder
Take CHARGE: Theater Testing of the Cultivating HeAlth Resilience Growth & Empowerment
TFV: tenofovir

## Data Availability

The datasets generated and analyzed during this study will be available from the corresponding author upon reasonable request.
